# Fevers: Their History, Etiology, Diagnosis, Prognosis and Treatment

**Published:** 1887-06

**Authors:** 


On Fevers: Their History, Etiology, Diagnosis, Prognosis, and Treatment. *By* Alexander Collie, M.D. (*Aberd.*), R. C. P. (*London*), *Medical Superintendent of the Eastern Hospitals. Pp. viii. and 288, 8 vo., with colored plates. Philadelphia:* P. Blakiston, Son & Co. *Chicago:* W. T. Keener, *1887.*

In his modest preface of eight lines, the author says, "The observations contained in this volume—Relapsing Fever, Rotheln and Influenza excepted—are for the most part founded upon over 21,000 cases which the author has personally treated from commencement to termination.

This little book can hardly be praised too highly. Chapter II, "On the treatment generally of acute infectious disease" is admirable. In common with all observers of very large experience, an experience involving thousands of cases, the author has learned that the use of drugs plays a very subordinate rôle in the treatment of acute infectious diseases.

The limits of a brief book-notice do not allow extended comment, but it is due this little volume to say that its perusal will well repay any medical man, for its quality and value are exceptionally high. E. W.

